# Habitat suitability analysis reveals high ecological flexibility in a “strict” forest primate

**DOI:** 10.1186/s12983-020-00352-2

**Published:** 2020-02-18

**Authors:** Malene Friis Hansen, Ventie Angelia Nawangsari, Floris M. van Beest, Niels Martin Schmidt, Mikkel Stelvig, Torben Dabelsteen, Vincent Nijman

**Affiliations:** 1 Research and Conservation, Copenhagen Zoo, Roskildevej 38, 2000 Frederiksberg, Denmark; 2 Behavioral Ecology Group, Department of Biology, University of Copenhagen, Copenhagen, Denmark; 3 Department of Biosciences, Aarhus University, Roskilde, Denmark; 4 Department of Social Sciences, Oxford Brookes University, Oxford, UK

**Keywords:** Abundance, Density, Distribution, *Macaca fascicularis*, Polyspecific association, *Trachypithecus auratus*

## Abstract

**Background:**

Research of many mammal species tends to focus on single habitats, reducing knowledge of ecological flexibility. The Javan lutung (*Trachypithecus auratus*) is considered a strict forest primate, and little is known about populations living in savannah. In 2017–2018, we investigated the density and distribution of Javan lutung in Baluran National Park, Indonesia. We conducted ad libitum follows and line transect distance sampling with habitat suitability analysis of Javan lutung.

**Results:**

Estimated density was 14.91 individuals km^− 2^ (95% CI 7.91–28.08), and estimated population size was 3727 individuals (95% CI 1979 – 7019). Long-tailed macaque (*Macaca fascicularis*) habitat suitability was the main driver of lutung habitat suitability as the probability of lutung occurrence increased greatly with macaque habitat suitability. Distance to roads, and distance to secondary forest had a negative relationship with lutung occurrence. Lutung habitat suitability decreased with increasing elevation, however, Mt Baluran and the primary forest on Mt Baluran was under-sampled due to treacherous conditions. Follows of six focus groups revealed considerable use of savannah, with terrestrial travel. The follows also revealed polyspecific associations with long-tailed macaques through shared sleeping sites and inter-specific vocalisations.

**Conclusions:**

Our study provides new knowledge on the general ecology of Javan lutung, such as use of savannah habitats, underlining our need to branch out in our study sites to understand the flexibility and adaptability of our study species. Another undocumented behaviour is the polyspecific association with long-tailed macaques. We encourage more research on this subject.

## Background

Studies on many tropical mammal species show a research bias towards specific habitats and often, single sites, thus severely restricting our notion of these species’ ecological flexibility and adaptability. This makes these studies potentially less relevant when comparing them to studies conducted on closely related species, to extrapolate to other areas, or to generalise to learn about broader topics. Chimpanzees (*Pan troglodytes*) for example, are often categorised by their forest behaviour, although their behaviour in savannah habitats differs greatly [1]. A study from 2013 reported many felids and primates using mangrove and peat swamp forest, yet most research conducted on them focused on their behavioural ecology in forests [2]. For other species, such as the leopard (*Panthera pardus*), research has focused on its behaviour in savannah areas, even though this behaviour is substantially different in forests [3]. Focusing on a single habitat reduces our knowledge of wildlife species, and leaves us at risk of creating misguided conservation initiatives, excluding potentially important habitats. We also end up undermining species adaptability and ecological flexibility, which again leads to misplaced conservation initiatives, such as relocating Bornean orangutans (*Pongo pygmaeus*) from areas we believe they cannot survive, even though this has never been investigated [4].

We here present data on a population of Javan lutung (*Trachypithecus auratus*), a colobine monkey endemic to the Indonesian islands of Java, Bali and Lombok that has been studied intermittently since the 1970s (Table 1). The only long-term research has been conducted at a rainforest site in western Java, Pangandaran, where annual rainfall exceeds 3000 mm and where there is a lack of distinct dry/wet seasons [5–10]. Shorter studies, typically up to 1 year in duration, have mainly been conducted in other rainforest sites (Mt Gede-Pangrango, 3000 mm: [11]; Mts Dieng, 4000 mm: [12]; Mt Bromo-Semeru, 3500 mm: [13]; Table 1). Especially in the eastern and northern part of their range, Javan lutung also occur in drier habitats, including mangroves, deciduous woodlands, teak (*Tectona grandis*) plantations, and savannah landscapes [14–17]. Compared to the wetter habitats, Javan lutung in these drier areas live in higher densities and larger group sizes [15], and may be ecologically distinct from their congeners in rainforests.

**Table 1:** Studies conducted on Javan lutung, with a general description of the main habitat type and amount of rainfall, showing that the majority of studies were conducted in areas with an annual rainfall over 2000 mm covered in rainforest. In bold are areas where one or more individual studies of more than 1 year of duration were conducted (excluding the present study)

Study site, province	Studies (n)	Years of publication	Annual rainfall (mm)	Habitat type
**Pangandaran, W Java**	16	1983–2019	3322	Lowland rainforest / forest plantation
**Mts Dieng, C Java**	4	1998–2018	4300	Lowland to montane rainforest
Halimun, Banten	5	1992–2008	3869	Hill to montane rainforest
Ujung Kulon, Banten	3	1972–1994	3519	Lowland rainforest
**Mt Gede-Pangrango, W Java**	3	1995–2013	3041	Hill to montane rainforest
**Mt Bromo-Tengger, E Java**	3	2008–2017	3500	Hill to montane rainforest
Mt Merapi, C Java	2	2014–2016	3675	Hill to montane rainforest
Mt Slamet, C Java	2	2012–2017	4280	Hill to montane rainforest
Mts Yang, C Java	2	2011–2013	2279	Montane rainforest
**Cepu, C Java**	2	1991–1994	1800	Deciduous forest / forest plantation
**Bali Barat, Bali**	2	2003–2013	1500	Deciduous forest
Mt Masigit-Kareumbi, W Java	1	2016	1900	Montane rainforest
Cikepuh, W Java	1	1994	3450	Lowland rainforest
Muara Gembong, W Java	1	1988	1650	Mangrove forest
Mt Ciremai, W Java	1	2014	3138	Hill to montane rainforest
Baluran, E Java	1	1986	1050	Deciduous forest

Predation pressure in open habitats differs from that in three-dimensional rainforests. While in some instances the same, or very similar, predators are present in both habitats, for arboreal primates the number and type of escape routes available when detected by a predator are distinctly smaller in open habitats. While it has been long acknowledged that gathering in polyspecific associations is one way to lessen predator pressure, it has been noted that for primates polyspecific associations are much rarer in Asia than in South America, Africa and Madagascar [18]. While polyspecific associations have not been documented with other primates, studies have documented associations between colobine monkeys and deer (e.g. [19]: *Semnopithecus entellus* and *Axis axis*, [20]: *T. auratus* and *Russa timorensis*), focusing on gleaning, where langurs drop plant matter, and deer subsequently consume it [19]. In West Bali National Park, Javan lutung and long-tailed macaques (*Macaca fascicularis*) have been observed within proximity of each other [21, 22], yet only agonistic encounters have been documented [21]. As of yet, there is no empirical evidence of a polyspecific relationship between Javan lutung and long-tailed macaques in the scientific literature.

Our main aim is to investigate the general ecology of Javan lutung in dry habitats, thus expanding our understanding of the habitat and conditions the Javan lutung lives under. We expect the Javan lutung to exhibit a higher ecological flexibility than previously assumed and thereby being able to exploit a diverse array of habitats, here also savannah. We investigate this by providing density and abundance estimates from within Baluran National Park (BNP), and quantifying habitat preference. Our methods include line transect distance sampling (LTDS), a method that has proven very successful for surveying mammal species, including primates [23–26], and Species Distribution Modelling (SDM), which quantifies wildlife habitat suitability. This method is also useful for conservation planning [27–29]. Our results will enable BNP officials to adopt scientifically based management and conservation initiatives for Javan lutung. Finally, our study provides baseline information that could be used in population trend assessments of the Javan lutung in other areas with similar habitats. Investigating the ecological flexibility of Javan lutung also includes looking at interactions with sympatric species, here the long-tailed macaque. We expect a high ecological flexibility to also include polyspecific associations, which if true will reveal a great adaptability of the Javan lutung to a very different environment, and discuss a behaviour not often experienced in Asian primates.

## Results

### Habitat use of Javan lutung

The six focal lutung groups ranged from eight to 20 individuals, and they used different habitats between and within groups (Table 2). We observed lutung groups foraging and travelling through different habitats, including anthropogenic habitats, as well as travelling and foraging on the ground in the savannah. We did not observe them consuming anthropogenic food items, or interacting with people.

**Table 2:** Often sighted Javan lutung groups and their sympatric long-tailed macaque groups (best-estimated mean group size)

Javan lutung groups with group sizes	Long-tailed macaque groups with group sizes	Shared sleeping habitat
Bekol, 15 (2 males)	Bekol, 121	Restored savannah, office and tourism area
Acacia^a^, 8 (1 male)	River, 35 Acacia, 66	Secondary forest Secondary forest
Bama^b^, 10 (1 male)	Acacia, 66 Bama, 88	Secondary forest Beach forest
Manteng^c^, 15 (2 males)	Manteng, 45	Beach forest
Batu Hitam, 10 (1 male)	Batu Hitam, 30	Beach forest
Mangrove, 20 (2 males)	Mangrove, 60	Mangrove

^a^Lutung group Acacia alternated between travelling/foraging with macaque group River and Acacia, yet shared sleeping site with Acacia

^b^Lutung group Bama alternated between travelling/foraging with macaque group Acacia and Bama. They shared sleeping area with macaque group Bama

^c^Lutung group Manteng alternated between travelling/foraging with macaque group Bama and Manteng. They shared sleeping area with macaque group Manteng

### Density and population size estimation

We encountered 233 individual lutungs in total including both outbound and return trip. Of the 233 individuals, all adults were black, no erythristic adult individuals were observed. The five infants still had their yellow natal pelage colour.

The estimated density of Javan lutung in BNP was 14.90 individuals km^− 2^ (95% CI 7.91–28.08 individuals km^− 2^; SE 4.87) (Table 3). The estimated mean group size was 2.92 individuals/sub-group, and estimated group density was 5.10 sub-groups km^− 2^. The total number of lutungs present in BNP was estimated at 3727 individuals (95% CI 1979–7019 individuals; SE 1217) (Table 3).

**Table 3:** Density and abundance estimates for Javan lutungs from Distance 7.1, half normal key, 2 cosine adjustments, 90 m truncation, AICc

Parameter	Estimate	SE	%CV	df	95%CI
ER	0.46	–	17.14	20	0.32–0.66
p	0.50	–	21.00	35	0.33–0.77
DS	5.10	1.38	27.10	55	2.99–8.70
E(S)	2.92	0.53	18.21	28	2.02–4.23
D	14.9	4.87	32.65	82	7.91–28.08
N	3727	1216.9	32.65	82	1979–7019

*ER* encounter rate, p: detection probability, DS: estimate of density of sub-groups (number per km^2^), E(S): estimate of expected value (mean) of sub-group size, D: estimate of density of individuals (number per km^2^), N: Abundance estimate, *SE* standard error, *CV* coefficient of variation, *df* degrees of freedom, and *CI* confidence interval

### Habitat suitability

The MaxEnt algorithm had an AUC = 0.82 and a TSS = 0.55, indicative of sufficient predictive capacity.

Four covariates had a variable importance higher than 0.05: macaque suitability (0.42) (positive relationship with lutung occurrence), distance to roads (0.09) (positive), distance to secondary forest (0.08) (positive), and elevation (0.06) (negative) (Figs. 2 and 4). Distance to rivers (positive), distance to restored savannah (positive), distance to savannah (positive), distance to shrub forest (positive), distance to trails (positive) and distance to agriculture and rice fields (negative) had the lowest variable importance (all > 0.05) for lutung habitat suitability (Fig. 4 and Appendix Fig. 1).

With increasing macaque habitat suitability, lutung probability of occurrence also increased until a macaque habitat suitability above 0.8, where the lutung probability of occurrence reached above 90% and remained stable (Fig. 2). On roads, there was no probability of lutung occurrence. Around 1 km away from roads the probability of encountering lutung drastically increased to 30% and then slowly increased until reaching 90% probability of occurrence at 8 km and onwards to 15 km distance from roads (Fig. 2). Inside secondary forest there was a 40% chance of encountering lutung, this then decreased until increasing again at 1 km distance from secondary forest. From 2.5 km onwards, there was a 90% probability of lutung occurrence. At approximately 50 m above sea level (masl), the chance of encountering lutung was 25%. Below 50 and above 50 masl the chance decreased drastically. From 800 masl, there was a very low probability of lutung occurrence.

The MaxEnt habitat suitability map (Fig. 3) revealed the highest suitability (0.8) in the secondary forest belt at low elevations surrounding Mt Baluran and in the area with invasive acacia and restored savannah near the tourist sites Bekol and Bama, as well as primary forest and savannah (Fig. 3).

### Co-occurrence and polyspecific associations with long-tailed macaques

During most of the focal group encounters (> 90%) in the early morning (prior to 07.30 h) and late afternoon (after 16.30 h), we also encountered long-tailed macaques within 50 m and 5 min, often within a few meters. During midday encounters and follows, we sporadically observed long-tailed macaques in close proximity to Javan lutung; in about half of the time, this was at foraging sites. This was a re-occurring and general pattern observed throughout the study period. There was a strong spatial overlap between individual long-tailed macaque groups and individual Javan lutung groups (Table 2). Out of 64 encounters on the transect lines in the line transect distance sampling census, including both outbound and return trips, 15 included long-tailed macaques within 50 m and 5 min. This provides us with a rate of mixed species spatial co-occurrence of 24%.

## Discussion

We report on what is one of the larger populations of lutung in Java showing use of secondary forest and savannah habitats. The density estimate was moderate when compared to those reported from rainforest sites such as Pangandaran (~ 15 individuals km^− 2^ vs ~ 190 individuals km^− 2^: [5, 8]) and Mts Dieng (23 individuals km^− 2^: [12]). Densities in BNP were double that of nearby West Bali National Park (~ 7 individuals km^− 2^: [22]). Unfortunately, differences in methodologies employed by various researchers (e.g. random placement of transects, section of study areas, team size, speed) hamper direct comparisons. The 95% CI for both density and abundance were large. Our observation size was low, and even though transects were placed throughout the park, extrapolating our transect results to the entire park may have resulted in the large confidence intervals. Transects only reached the base of Mt Baluran, yet we still included it in our estimates because we know lutung inhabit the mountain [pers. comm. Arif Pratiwi]. Conducting the census again with transects or points on the mountain may reduce the confidence interval.

Group sizes of lutung recorded in BNP, i.e. eight to 20 individuals, appear to be typical for the species [14, 30, 31], and did not seem to differ considerably between habitats (Table 2). However, our three smallest groups (Acacia, Bama, and Batu Hitam) all used savannah habitats during daytime. This contradicts findings of Nijman [15], who, as part of an island-wide survey documented larger group sizes in drier habitats. For all groups we experienced them in several different habitats with a very clear temporal pattern, only obstructed by visitor presence, as all lutung groups avoided visitors as much as possible.

**Fig. 1:**
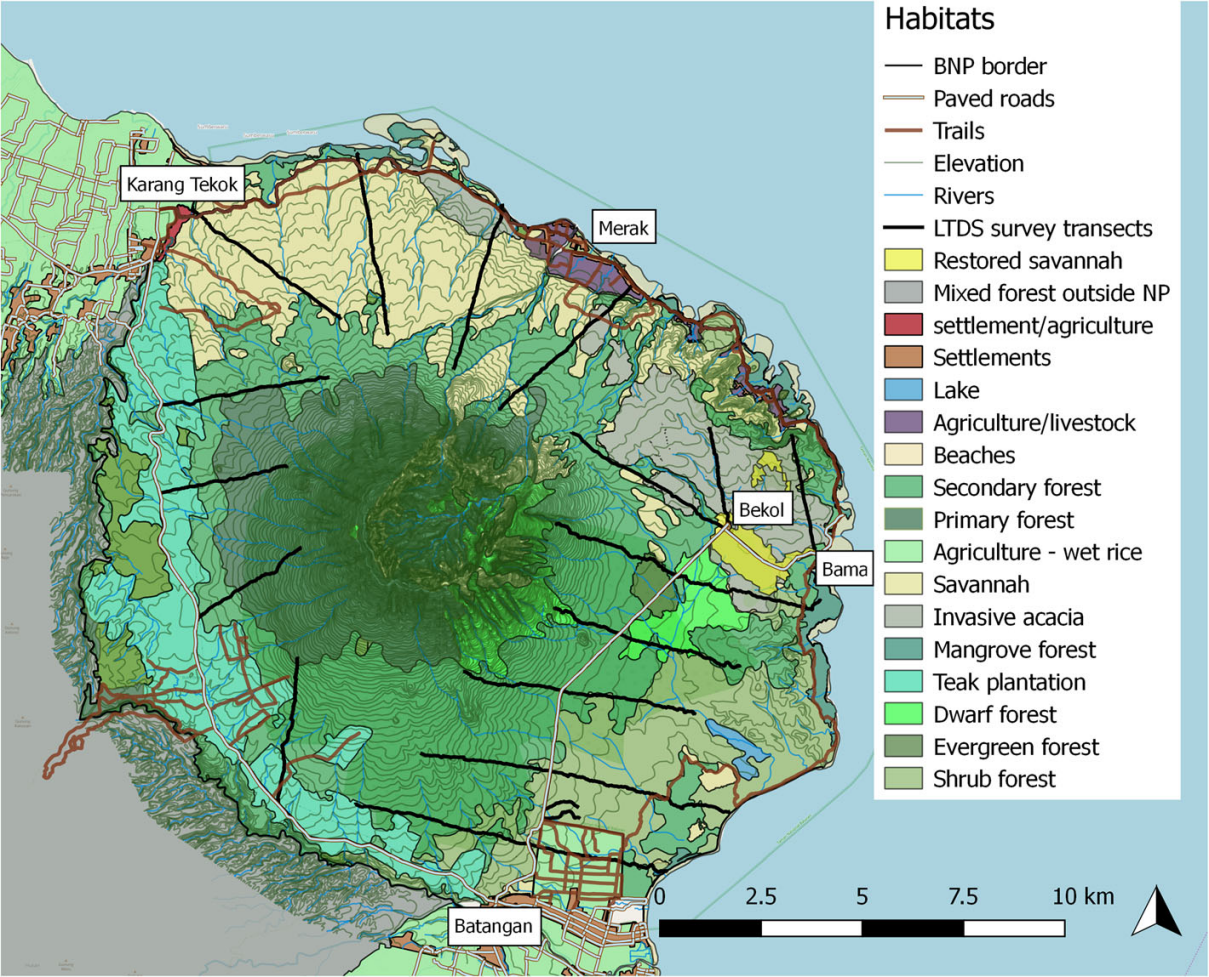
QGIS map of Baluran National Park with transects, and habitat types. Surroundings consists of villages and wet rice (South and Northwest), the Bali Strait (East) and unprotected mixed forest and agriculture (West)

**Fig. 2:**
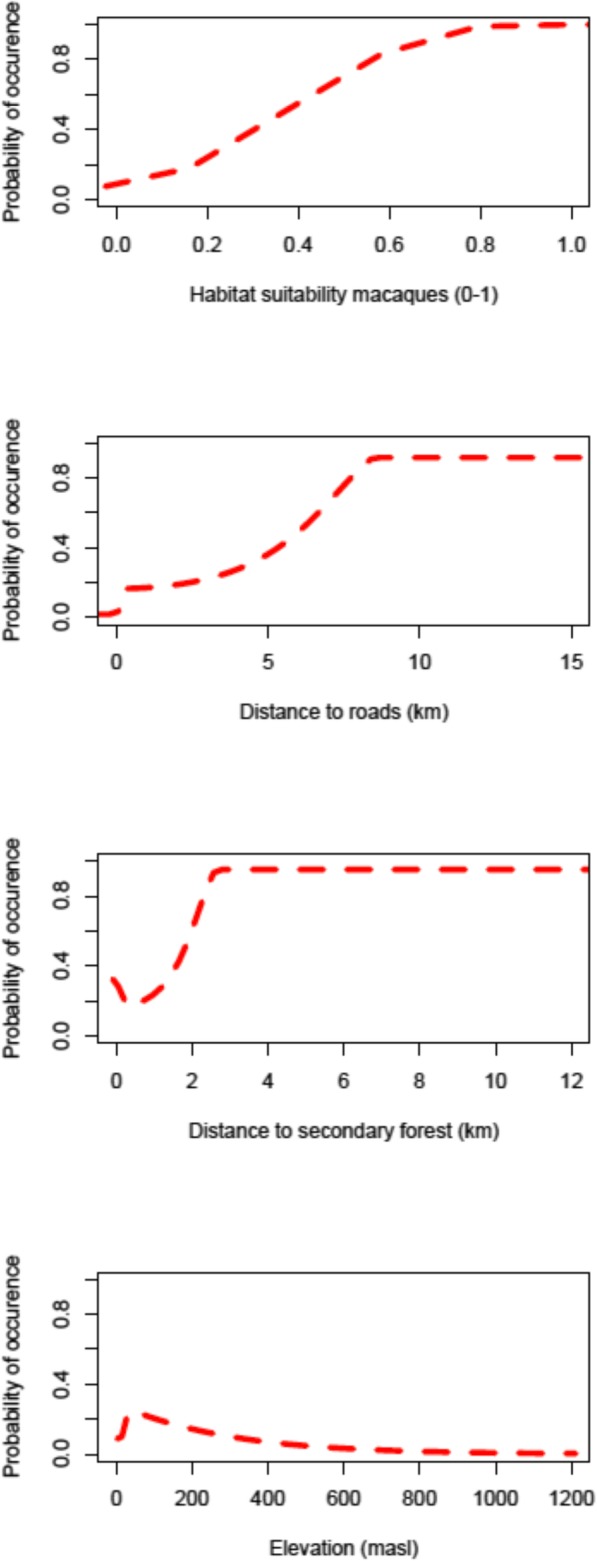
Response curves for the four variables with highest importance (out of the 10 included covariates) as based on the MaxEnt algorithm. As shown, the probability of lutung occurrence increased with increased habitat suitability of macaques, with increased distance to roads and with increased distance to secondary forest, while it decreased with increased elevation. Although of lower variable importance, the probability of lutung occurrence also increased with increasing distance to shrub forest, restored savannah, savannah and rivers, while the probability of lutung occurrence decreased with increasing distance to agriculture and rice fields (Appendix Fig. 1)

**Fig. 3:**
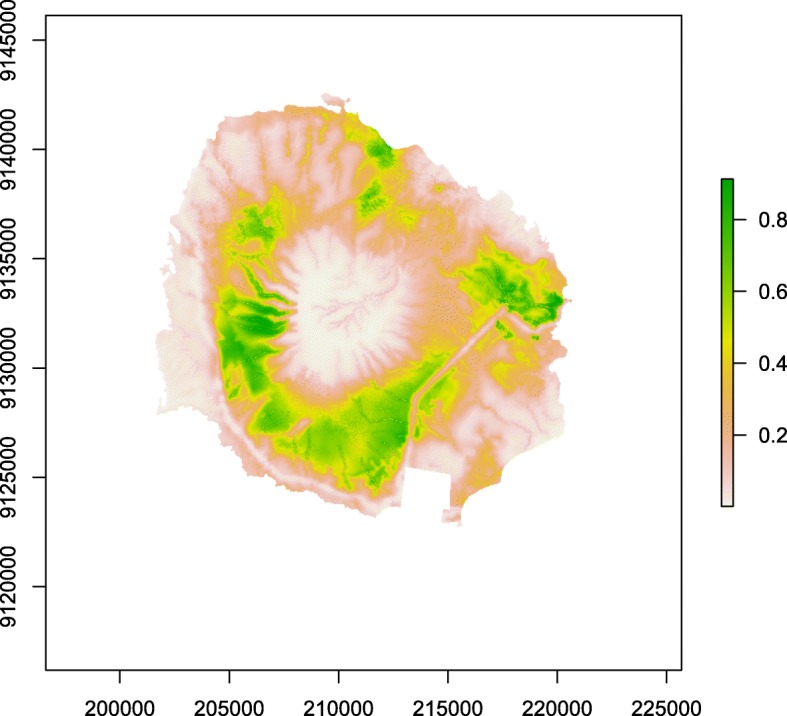
MaxEnt habitat suitability map. The legend shows lutung habitat suitability ranging from high (green) to low (white). The x and y-axis show UTM Easting and Northing. Primary forest on Mt Baluran was under-sampled due to treacherous conditions

**Fig. 4:**
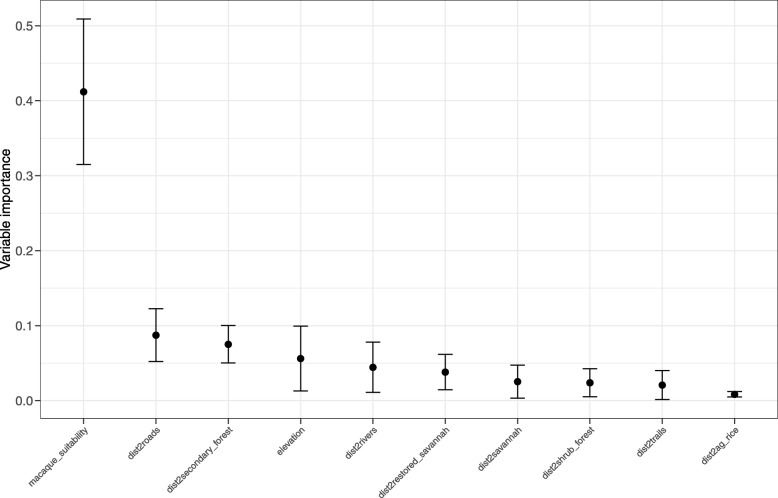
Environmental variable importance based on the MaxEnt algorithm. The mean variable importance is shown as a dot with associated 95% confidence intervals as bars, which were calculated based on 10 MaxEnt runs using a bootstrap procedure (see text for details)

Ad libitum follows showed intergroup habitat variation, yet no general pattern for the species of Javan lutung. However, environmental variable importance (Fig. 4) revealed that lutung occurrence clearly followed macaque suitability (0.42 importance) (Fig. 4). This indicates that the main driver for lutung occurrence is macaque occurrence, and the pattern for distribution follows macaque distribution. The main driver for macaque distribution was distance to roads and trails, yet in the opposite direction of the lutung, with probability of occurrence decreasing with increasing distance to roads and trails [26]. The habitat suitability map (Fig. 3) shows that roads are of low suitability, mostly below 0.2, yet areas around roads had high suitability (up to 0.8). Lutung and macaques seem to be present in the same habitats, yet the shyer lutung does not appear directly on roads. Indeed, increasing distance to roads increased the probability of lutung occurrence (Fig. 2). However, the small size of BNP leaves it difficult to be 15 km away from roads. The small spike in probability of occurrence at 1 km distance to roads fits the habitat suitability map, and is probably the most important indicator of the relationship between lutung occurrence and distance to roads. In the more western parts of its range Javan lutung have been observed in forests adjacent to smaller roads but not main roads [Mts Dieng, pers. obs. V. Nijman], and to use trees lining larger roads in late afternoon when few people and cars are around [Pangandaran, pers. obs. V. Nijman]. The probability of Javan lutung occurrence in secondary forest was approximately 40% inside secondary forest and 90% from 2.5 km and outwards. Again, the most important indicators are found at low distances because of the size of BNP. The secondary forest in BNP is situated around the bottom of Mt Baluran. It contains mostly mixed deciduous forests and open woodland. Encroachment of this part of the national park is lower than most other habitats, and this may be why it is a preferred habitat. The heterogeneity of habitats in BNP is high (Fig. 1) and therefore surrounding habitats of secondary forests includes almost all habitat variables and as such the habitats of high probability of occurrence of Javan lutung (90%) 2.5 km away from secondary forest and onwards are hard to determine.

Elevation was one of the four covariates with highest importance for lutung habitat suitability (0.06 importance). We saw a decrease in lutung occurrence with elevation increase; however, there was an increase in probability of occurrence until 50masl, after which the probability dropped. BNP is mostly situated at sea level, and only Mt Baluran reaches above 50masl, yet we did not include Mt Baluran in our census grid and this will have affected our results somewhat. Throughout Java, Bali and Lombok, Javan lutung are found in the montane and upper-montane regions up to 3500masl [14]. Mt Baluran (1250masl) is covered in primary forest, and the Javan lutung was found to be present on the mountain during our period of research (pers. comm. Arif Pratiwi). If Mt Baluran was included, possibly lutung occurrence along the elevational gradient may have differed slightly than found in our models, possibly also resulting in primary forest becoming a covariate of importance to lutung habitat suitability.

We observed Javan lutung in savannah and restored savannah, and some of these areas have high suitability (0.8) in the habitat suitability map (Fig. 3). One of these areas is situated close to areas with high macaque suitability, the tourist sites of Bekol and Bama (Fig. 1) [26]. Another is in the large Merak savannah, which did not have high suitability for macaque suitability [26]. This area does not contain any roads, and that may reflect the difference in habitat suitability between the two species. We observed the Javan lutung foraging and travelling on ground, which to our knowledge is a very novel observation.

The habitat suitability map (Fig. 3) can be used for creating management plans for the Javan lutung. It shows the habitat presence of Javan lutung with several high preference areas (0.8 and above). Macaque habitat suitability had much higher importance for lutung habitat suitability than any distance to covariates, emphasizing the polyspecific associations we observed between the two species. Other covariates may be of importance to lutung habitat suitability, such as predator density, sympatric species distribution, and food availability and quality, and we recommend incorporating these in future SDM habitat suitability studies of Javan lutung.

The co-occurrence between lutungs and long-tailed macaques has been rarely recorded in other study sites. Almost all encounters with lutungs in the early morning and late afternoon were accompanied by long-tailed macaque groups, and according to our observations regarding group size and composition, as well as travelling patterns, the same lutung groups had close proximity with the same long-tailed macaque groups. The co-occurrence of Javan lutung with long-tailed macaques was so predictable that long-tailed macaque groups were used to find lutung groups and vice versa for long-tailed macaques research [26], especially in morning and afternoon. Both species were observed to react to vocalisations of each other, especially alarm calls. We did not observe agonistic encounters between the two species, yet we did experience them foraging together and travelling together. These observations were not chance encounters, and as such could suggest that Javan lutungs and long-tailed macaques may have polyspecific associations in BNP. Indeed, shared sleeping trees and joined travelling can increase predator surveillance and avoidance. BNP primate species are vulnerable to ground, tree and sky predators. Javan lutung individuals were in general, observed at a greater tree height than long-tailed macaques. Safety in numbers, as well as having surveillance for predators at different heights may increase protection from predators. The shared social information between two sympatric species (such as vocalisation and travelling) can increase predator avoidance and thereby survival even in two seemingly competing species, especially when trying to avoid a generalist predator, and especially in low density areas [32]. The Javan lutung and the long-tailed macaques were mostly observed together away from roads and in non-tourist areas, where long-tailed macaque groups have lower densities (< 100 individuals), and they are both prey of the generalist predator; the Javan leopard (*Panthera pardus melas*) [33, 34]. Gurmaya et al. [31] conducted line transect surveys in Ujung Kulon National Park and in 14% of their encounters lutung and long-tailed macaques were observed in close proximity. The high densities of both lutungs and long-tailed macaques in Pangandaran [pers. obs. V. Nijman] makes it inevitable that their ranges largely overlap, but interactions as observed in BNP appear to be less common elsewhere e.g. [35].

The spatial co-occurrence of Javan lutungs and long-tailed macaques for our qualitative observations were high (> 90%) for early morning and late afternoon encounters. For midday observations, it resembled the distance sampling co-occurrence of 24%. This is twice as high as reported for West Bali National Park [22]. Javan lutungs were also observed responding to long-tailed macaque vocalisations in West Bali National Park [22]. We did not see any patterns in habitat choice for spatial co-occurrence for Javan lutungs and long-tailed macaques, except for sleeping habitats (Table 2). It is difficult to conclude if what we saw is mutualism or random association around mutual resources, however, the high co-occurrence and the clear temporal association pattern indicates a polyspecific relationship, where both species seem to have adapted to each other. Previous studies suggest gleaning from sympatric wildlife [18, 20], yet this was not observed in our study.

## Conclusions

Our study provides much needed information regarding the Javan lutung in dry habitats. We provide density estimates from a systematic census, observations of high ecological flexibility, and observations of a possible polyspecific relationship between two Asian primates not documented before. We recommend that our study is continued, and especially the line transect distance sampling census conducted yearly to improve resolution and reduce the estimate variation, as well as allowing park management to follow the population fluctuation over time [36]. In long-term field sites, such as Baluran National Park, monitoring primate populations should be a regular activity [37]. We also recommend surveys on polyspecific associations in other parts of the Javan lutung range where sympatry with long-tailed macaques occurs.

The Javan lutung are able to exploit many different habitats, even dry habitats, and habitats with disturbance without interacting with humans or engaging in conflicts with humans. This underlines their ecological flexibility, and the need for researchers to study them across their range in many different habitats. However, as habitats are encroached further, Javan lutung may not be able to continue to adapt without interacting with humans, or becoming extirpated. We underline that our habitat suitability map can aid greatly in creating informed conservation action plans both for BNP, yet also for other areas. Especially West Bali National Park that has experienced a reduction in Javan lutung population size [22] may be able to reproduce our census, and even use our results, as habitats are similar. Researchers have neglected the great ecological flexibility of Javan lutung so far. We recommend that future conservation initiatives take a broader approach to Javan lutung conservation, and acknowledge important Javan lutung habitats, including those with little canopy cover, where terrestrial travel between trees is the only option, and where cooperation with other primate species may help against ground predators. Including environmental parameters such as precipitation and elevation together with sympatric primate habitat suitability and other habitat variables in habitat suitability analysis across Javan lutung range would provide an important insight into their ecology and explore their ecological flexibility even further.

Our results show the necessity of expanding research to include surrounding habitats of our study species. When we study wildlife, we may identify preferred habitats; however, this does not mean that the species in question are not able to exploit other habitats, or adapt their ecology to changing habitats. Expanding our research will expand our knowledge and enable us to create knowledge-based conservation initiatives. We expected the Javan lutung to exhibit high ecological flexibility, and we proved that it indeed did, with savannah occurrence, terrestrial travel and a polyspecific association with long-tailed macaques. We recommend considering our results in future research and conservation of the Javan lutung.

## Methods

### Study site

Baluran National Park (BNP) is located on the northeastern tip of Java (7°50′0″S, 114°22′0″E) in Indonesia. The total area is 250km^2^ and includes primary and secondary forest, savannah, shrub forest, and mangroves (Fig. 1). Two species of primates are currently found in the national park, the Javan lutung and the long-tailed macaque, while the Javan slow loris (*Nycticebus javanicus*) may occur in parts yet to be surveyed [38]. Potential predators of the primate species include the Javan leopard, dhole (*Cuon alpinus*), various birds of prey such as the changeable hawk-eagle (*Nisaetus cirrhatus*) and short-toed snake-eagle (*Circaetus gallicus*), and the reticulated python (*Malayopython reticulatus*).

Human activity within the park influences the biodiversity, especially mammal species richness in areas surrounding permanent settlements [39, 40]. These settlements were already established when the park was gazetted in 1980 [40], and their approximately 4000 domestic cows and goats use 22% of national park habitat for grazing, which has had a negative impact on native mammal wildlife occurrence in the area [39]. Native wildlife in BNP is also threatened by invasive acacia (*Acacia nilotica*), that has invaded the native savannah, and in 2013 covered roughly 90% of it [41, 42]. Tourism is extensive in designated areas (86,000 visitors in 2017) [pers. comm. Arif Pratiwi], and a highly trafficked highway traverses the outer southern part of the park.

### Study species

The Javan lutung is a diurnal colobine primate that occurs only in Indonesia; on the islands of Java, Bali and Lombok [43]. It is classified as Vulnerable on the IUCN Red List with a decreasing population size [43]. The Javan lutung diet is more versatile than other folivorous colobines, and includes young leaves, fruits and flowers. They are able to also feed on the leaves of teak trees (*Tectona grandis*) in plantations [8]. They occur at different elevations ranging from 0-3500masl [14], and experience lower densities and group sizes at higher elevations [15]. Information on home ranges is only available from the long-term field site in Pangandaran; here Javan lutung groups have well-defined home ranges of ~ 4-6 ha [8].

### Data collection

On 2 December 2019, we conducted a search in Google Scholar for articles written about Javan lutung to assess if there had been a study bias to one or more particular habitat types (Table 1). We used ‘*Trachypithecus auratus*’, ‘*Presbytis auratus*’ and ‘*Presbytis aurata*’ as search terms, and included only studies that were conducted on wild populations (thus excluding studies on captive lutungs) that were conducted at one location (thus excluding surveys). We included articles, reports, book chapters, and theses in English, German and Indonesian.

#### Follows and ad libitum observations

Research was conducted from February 2017 through June 2017 for preliminary observations, liaisons with stakeholders and reconnaissance, spending approximately 3 days per week inside BNP, and from July 2017 to May 2018 for more systematic data collection on the primates, spending 5 days per week inside BNP following focal groups or conducting line transect distance sampling (see below). Wet season in BNP occurs from November through April, and dry season from May through October with modifications each year. We conducted follows of groups at different time slots during the day, divided into morning (05.30–09.00 h), midday (09.00–15.00 h) and afternoon (15.00–17.30 h), and this was augmented by ad libitum observations [44]. The lutungs were mostly located by vocalisations and sounds from shaking branches and leaves as the animals move through the canopy. None of the groups were habituated. We followed Javan lutung groups from the sleeping sites into other habitats as far as they and the landscape permitted in the morning, tried to locate them midday and follow them back to their sleeping sites in the afternoon, with detection certainty and following distance decreasing with habituation. We encountered six groups regularly. They were identified by the location of their sleeping sites, their group sizes and composition, especially the number of adult males (Table 2). Javan lutung groups have well-defined small home ranges in Pangandaran ~ 4-6 ha [8], and we expect this to be the case in BNP as well, although habitats differ. For observations, we maintained a distance of 20 m in areas such as sleeping sites, whereas in the dense scrub habitats in BNP this distance increased greatly until the groups were not visible anymore.

#### Line transect distance sampling

We created a systematic grid covering the entire area, excluding the higher parts of Mt Baluran (200masl – 1250masl), which was too treacherous for a systematic census. This did however; exclude a large part of the primary forest, which could have affected our results. Because Javan lutung are the most observed mammal species on the mountain [pers. comm. Arif Pratiwi], we decided to include the mountain in our estimates. We placed the grid randomly in respect to habitats and wildlife distribution [25, 45], but ensured all transects began at roads or trails (Fig. 1). This ensured that we did include all habitats, also anthropogenic ones. We did not place transects according to anecdotal knowledge of lutung distribution to ensure that we did not affect results through our transect grid.

All transects were 4.5 km long with at least 2.0 km between adjacent transects totalling 189.0 km for outbound and return trip. However, we were only able to walk 160.65 km due to inaccessibility (Fig. 1). Between October and December 2017, a team of three to four observers walked the transects at a speed of 1.25 km hr.^− 1^, finishing one transect trip (outbound or return) within 4 h with observations never exceeding 15 min [24, 25]. Outbound trips were conducted in the morning between 07.00 and 11.00 h, and return trips in the afternoon between 13.00 and 17.00 h. An observation was the detection of Javan lutung, repeated counts, and distance measurement. We aimed at detection of all Javan lutung at zero distance (on the line) [25, 46]. To keep our bearing and direction, find the perpendicular point on the transect (initial location of detection) and waypoint the position, and measure the perpendicular distance (PD) to sub-group centre (i.e. the midpoint of individuals that were within sight), we used a Garmin GPS Map 64 s, a compass, and a Nikon Aculon A11 Rangefinder [23, 38, 47]. We used repeated counts to count sub-group size [23]. To increase our resolution of distance measurements, we focused on smallest visible clusters of individuals – “sub-groups”, where group centre is more accurately estimated [36, 48]. We always measured the PD from the line [25]. We detected animals via sight and vocalisations, yet only counted them when visual [37]. We recorded the pelage colour of Javan lutung, distinguishing between black and erythristic individuals.

#### Co-occurrence and polyspecific associations with long-tailed macaques

When searching for Javan lutung, we also systematically searched the surrounding landscape for long-tailed macaque groups, and registered all interactions. All encounters of long-tailed macaques were also registered during line transect distance sampling. We recorded encounters within 50 m and 5 min of a Javan lutung group as a spatial co-occurrence encounter [22].

### Data analysis

#### Line transect distance sampling

We only included the outbound trip (41 sub-group sightings) in our analysis to avoid double counts. The analysis was conducted in Distance 7.1 [49]. For calculating population size, we considered the entire BNP area, i.e. 250km^2^, acknowledging that lutung also range outside BNP, especially to the southwest towards Mt Ijen [14]. We could not secure accurate PD for three sightings, and these were excluded from the analysis.

We right-truncated our data to increase robustness in estimating detection function, excluding all observations beyond 90 m [24]. We investigated histograms without truncation to determine truncation distance [pers. comm. Eric Rextad and Tiago Marques]. This excluded four observations, approximately 10% of the data [24, 49]. We tested all detection function combinations: 1. Uniform key with cosine, simple polynomial, and hermite polynomial adjustments, 2. Half-normal key with cosine, simple polynomial, and hermite polynomial adjustments, 3. Hazard rate key with cosine, simple polynomial, and hermite polynomial adjustments. Due to lowest AICc and best goodness of fit (GOF) tests according to *p* < 0.05 and lowest χ^2^/df accumulated for χ^2^ tests half-normal key detection function with 2 cosine adjustments was chosen [22, 24, 46]. We used the lowest AICc due to the low observation size [24].

#### Species Distribution Modelling (SDM) environmental predictors

Shapefiles containing vector layers provided by BNP, updated using Google Earth enabled us to create a map of BNP in QGIS 2.18.19, in which vector layers from the LTDS census were inserted. Using the raster package in R [50], we generated 26 raster layers (15 × 15 m resolution) with potential environmental predictor variables for the habitat suitability analyses (described below). Topographic layers (*n* = 5) included elevation (m), slope (°), aspect (*radians*), hill-shade (*radians*) and terrain ruggedness (index), derived from a digital elevation model of the study area. We generated a raster layer using ESRI shapefiles, that included 13 major vegetation/habitat types found in the census region including: rice fields, livestock fields, teak plantations, dwarf forests, evergreen forest, shrub forest, primary forest, secondary forest, mangroves, acacia forest, savannah, restored savannah, and beach. Instead of using categorical classes for each habitat/vegetation type, and to capture potential edge effects in habitat suitability, we generated new rasters (15 × 15 m pixel size). We did this by calculating for each raster cell the Euclidean distance (km) to the nearest cell with a given vegetation type (*n* = 13). Euclidean distance to (km) paved roads, human settlements, trails, and rivers found in the census region were included in the eight last raster layers.

Once all raster layers were created, we excluded collinear raster layers by calculating the Variance Inflation Factor (VIF), then excluding the one with highest VIF and repeating this process until only layers with a VIF < 2 were remaining [51]. With this procedure we ended up with 10 covariates for the final MaxEnt modelling; macaque suitability, distance to roads, distance to secondary forest, elevation, distance to rivers, distance to restored savannah, distance to savannah, distance to shrub forest, distance to trails and distance to agriculture and rice.

#### SDM evaluation and mapping

We used the SDM package in R for modelling and mapping of habitat suitability, and employed the MaxEnt algorithm [52]. Presence locations (*N* = 62) comprised projected waypoints of observations from both the outbound and return trip of each transect from the LTDS population census. All non-correlated environmental raster layers were used to extract the values of environmental conditions for presence points.

Model accuracy of MaxEnt was assessed by calculating the area under the curve of the receiver operating characteristic (AUC [53];). An AUC value of 1 indicates perfect performance, whereas an AUC value of 0.5 indicates that the model performs no better than a random model. AUC > 0.7 generally indicates good model accuracy [27]. We furthermore calculated the True Skill Statistic (TSS). A TSS value below 0 indicates a no better than random model performance, and a value of 1 indicates perfect performance [54]. Change in the AUC value (ΔAUC) with and without a specific environmental variable, but with all other variables included, was used to evaluate variable importance for the habitat suitability map.

## Supplementary information

**Additional file 1:.** Figure S1. the Javan lutung in Baluran National Park.

**Additional file 2:.** Figure S2. savannah in rainy season with Baluran Mountain in the background in Baluran National Park.

**Additional file 3:.** Figure S3. Long-tailed macaques in Baluran National Park.

**Additional file 4:** Appendix Fig. 1. Response curves for the six variables with lowest importance (out of the ten included covariates) as based on the MaxEnt algorithm.

## Data Availability

Data is available through Dryad Data Repository DOI:10.5061/dryad.cc2fqz638.

## Abbreviations

AICc: Akaike information criterion with corrections for small sample sizes
AUC: Area under the curve
BNP: Baluran National Park
CI: Confidence interval
CPH Zoo: Copenhagen Zoo
CV: Coefficient of variation
D: Estimate of density of individuals (number km^− 2^)
df: Degrees of freedom
DS: Estimate of density of sub-groups (number km^− 2^)
E: East
E(S): Estimate of expected value (mean) of sub-group size
ER: Encounter rate
ESRI: Environmental Systems Research Institute
GOF: Goodness of fit
GPS: Global Positioning System
ha: Hectare
hrs: Hours
IUCN: International Union for Conservation of Nature
km: Kilometre
LTDS: Line transect distance sampling
m: Meter
masl: Meter above sea level
MaxEnt: Maximum entropy
min: Minutes
mm: Millimetre
Mt: Mountain
N: Abundance estimate
n: Number
PD: Perpendicular distance
QGIS: Quantum Geographic Information System
S: South
SDM: Species distribution modelling
SE: Standard error
*T.*: *Trachypithecus*
TSS: True Skill Statistic
VIF: Variance inflation factor
