# Supplementary figures and images for: Habitat suitability analysis reveals high ecological flexibility in a “strict” forest primate
Source: Front Zool. 2020 Feb 18;17:6. doi: 10.1186/s12983-020-00352-2 (PMC7027213; doi:10.1186/s12983-020-00352-2)

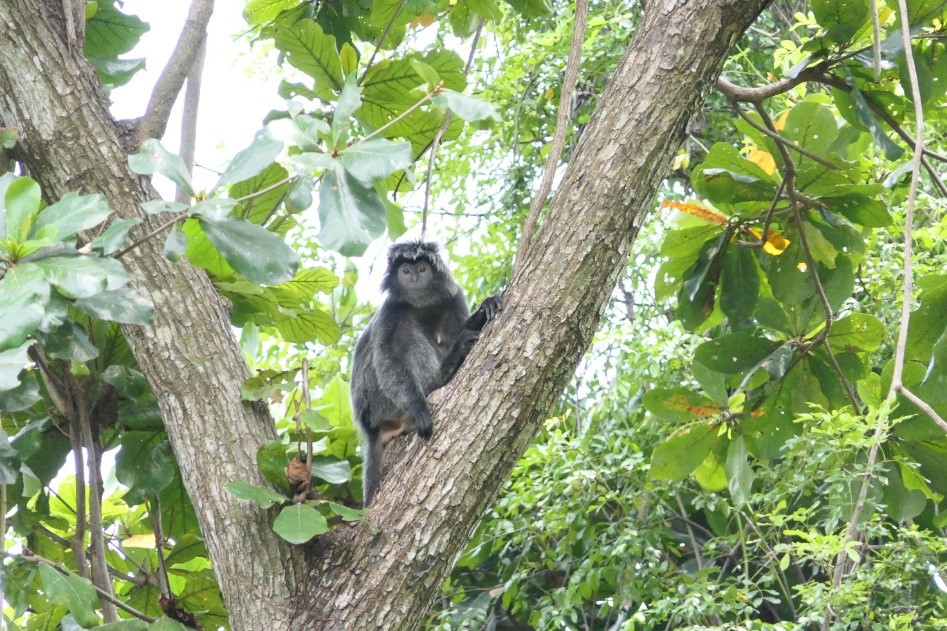

Supplement: Supplementary file 1 — Additional file 1:. Figure S1. the Javan lutung in Baluran National Park. [file 12983_2020_352_MOESM1_ESM.jpg]

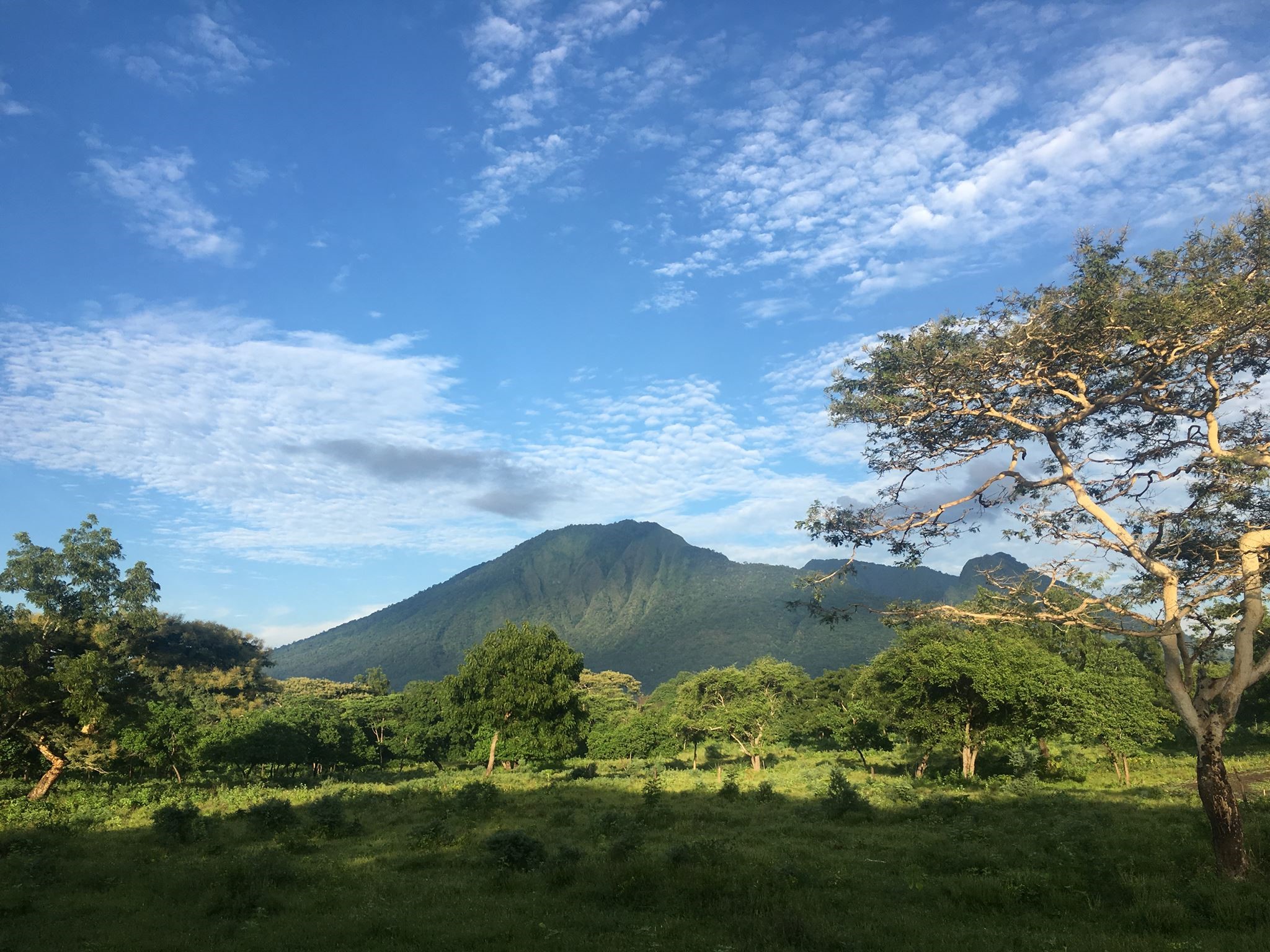

Supplement: Supplementary file 2 — Additional file 2:. Figure S2. savannah in rainy season with Baluran Mountain in the background in Baluran National Park. [file 12983_2020_352_MOESM2_ESM.jpg]

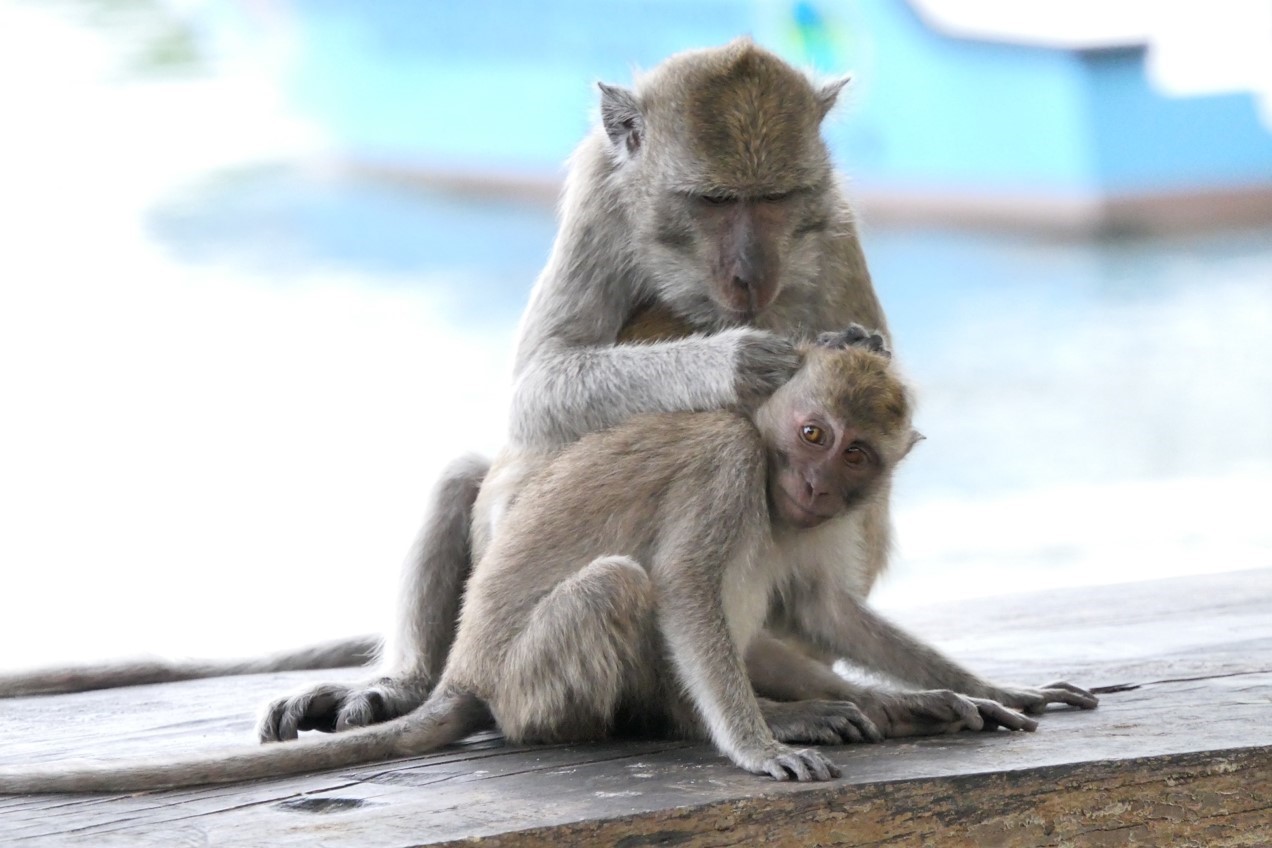

Supplement: Supplementary file 3 — Additional file 3:. Figure S3. Long-tailed macaques in Baluran National Park. [file 12983_2020_352_MOESM3_ESM.jpg]

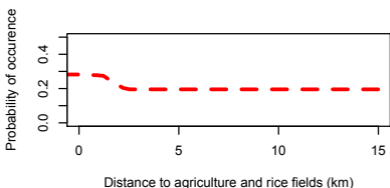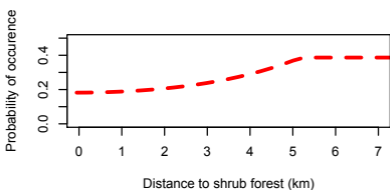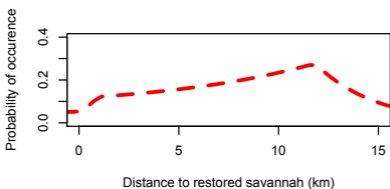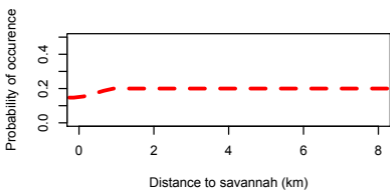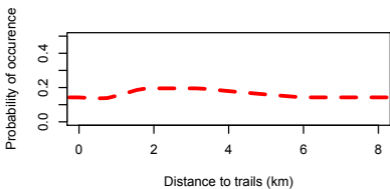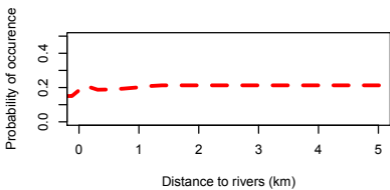

Supplement: Supplementary file 4 — Additional file 4: Appendix Fig. 1. Response curves for the six variables with lowest importance (out of the ten included covariates) as based on the MaxEnt algorithm. [file 12983_2020_352_MOESM4_ESM.pdf]
